# A Novel Mechanism Regulating Dopamine Receptor Type 2 Signal Transduction in Pituitary Tumoral Cells: The Role of cAMP/PKA-Induced Filamin A Phosphorylation

**DOI:** 10.3389/fendo.2020.611752

**Published:** 2021-02-16

**Authors:** Federica Mangili, Donatella Treppiedi, Rosa Catalano, Giusy Marra, Genesio Di Muro, Anna Spada, Maura Arosio, Erika Peverelli, Giovanna Mantovani

**Affiliations:** ^1^ Department of Clinical Sciences and Community Health, University of Milan, Milan, Italy; ^2^ Endocrinology Unit, Fondazione IRCCS Ca’ Granda Ospedale Maggiore Policlinico, Milan, Italy

**Keywords:** pituitary neuroendocrine tumors, filamin A phosphorylation, dopamine receptor type 2, cAMP/PKA pathway, signal transduction

## Abstract

The actin binding protein filamin A (FLNA) is required for somatostatin receptor 2 (SSTR2) and dopamine receptor 2 (DRD2) expression and signaling in GH- and PRL-secreting PitNETs, respectively, playing a role in tumor responsiveness to somatostatin receptors ligands and dopaminergic drugs. FLNA functions are regulated by several mechanisms, including phosphorylation. It has been shown that in GH-secreting PitNETs FLNA phosphorylation on Ser2152 (P-FLNA) switches FLNA function from a scaffold that allows SSTR2 signal transduction, to a signal termination protein that hampers SSTR2 antitumoral effects. Aims of the present study were to evaluate in PRL- and ACTH-secreting PitNETs cell lines MMQ and AtT-20 the effects of cAMP pathway activation and DRD2 agonist on P-FLNA and the impact of P-FLNA on DRD2 signal transduction. We found that forskolin increased (+2.2 ± 0.8-fold, p < 0.01 in MMQ; +1.9 ± 0.58-fold, p < 0.05 in AtT-20), and DRD2 agonist BIM53097 reduced (-49.4 ± 25%, p < 0.001 in MMQ; -45.8 ± 28%, p < 0.05 in AtT-20), P-FLNA on Ser2152. The overexpression of a phosphomimetic (S2152D) FLNA mutant in both cell lines prevented DRD2 antiproliferative effects, that were comparable in cells transfected with empty vector, wild-type FLNA as well as phosphodeficient FLNA mutant (S2152A) (-20.6 ± 5% cell proliferation, p < 0.001 in MMQ; -36.6 ± 12%, p < 0.01 in AtT-20). Accordingly, S2152D FLNA expression abolished the expected ability of BIM53097 to increase or decrease, in MMQ and in AtT20 respectively, ERK phosphorylation, an effect that was maintained in S2152A FLNA expressing cells (+1.8 ± 0.65-fold, p < 0.05 in MMQ; -55 ± 13%, p < 0.01 in AtT-20). In addition, the inhibitory effects of DRD2 on hormone secretion (-34.3 ± 6% PRL, p < 0.05 in MMQ; -42.8 ± 22% ACTH, p < 0.05 in AtT-20, in cells expressing S2152A FLNA) were completely lost in S2152D FLNA transfected cells. In conclusion, our data demonstrated that cAMP pathway and DRD2 agonist regulated FLNA activity by increasing or decreasing, respectively, its phosphorylation. Moreover, we found that P-FLNA prevented DRD2 signaling in PRL- and ACTH-secreting tumoral pituitary cell lines, suggesting that this FLNA modification might represent a new regulatory mechanism shared by different GPCRs. In PitNETs expressing DRD2, modulation of P-FLNA might suggest new pharmacological strategies to overcome drug resistance, and P-FLNA might represent a new biomarker for tumor responsiveness to dopaminergic agents.

## Introduction

Pituitary neuroendocrine tumors (PitNETs) are endocrine tumors that represent the 10–25% of all intracranial neoplasms classified on the base of their secretory activity. PitNETs may give rise to neurological manifestations, visual defects depending on mass spread effect, or endocrine syndromes related to hormonal hypersecretion. Although generally benign, infiltration of tumor tissue into near structures together with medical therapy resistance, are the main obstacles to their treatment (1, 2).

Dopamine receptor type 2 (DRD2) represents the principle target for pharmacological therapy with dopamine agonists (DAs) due to its ability to induce tumor mass shrinkage and to regulate excessive hormone secretion in prolactin (PRL)-secreting PitNETs (3).

Studies indicated DRD2 as potential target for other types of PitNETs, such as adrenocorticotrophic (ACTH)-secreting PitNETs (4–7), since up to 75% of them express functional DRD2 (8–10). ACTH-secreting PitNETs are characterized by chronic hypercortisolism and the treatment of choice is trans-sphenoidal surgery (11). Surgical failure occurred in most cases and pasireotide represents the only tumor-targeted drug approved for recurrent or persistent ACTH-secreting PitNETs, but it has not been proved effective for a widespread clinical use (12). Pharmacological treatment with DAs is successful in most patients with PRL-secreting PitNETs, but 10–20% present resistance to these medications (13, 14), probably due to low DRD2 expression (15, 16), alterations of Gαi2 inhibitory subunits mRNA levels (17), low amount of filamin A (FLNA) (18), DRD2 polymorphisms (19, 20) or unknown mechanisms.

Despite some clinical benefits have been shown (4, 8, 21), a strong support for the use of DAs in ACTH-secreting PitNETs is lacking.

The molecular mechanisms underlying pharmacological resistance have not been fully understood and no biomarkers predictable of response to medications are yet available.

In the last years, a role for cytoskeleton and its associated proteins in regulating receptors expression and signaling has been highlighted (22).

In particular, the actin binding protein FLNA regulates localization, expression and signaling of somatostatin (SS) receptor type 2 (SSTR2) and DRD2 in GH- and PRL-secreting PitNETs respectively, with important implications in pharmacological approach with SS receptor ligands (SRL) and DAs (18, 23–25). Indeed, FLNA silencing prevented the inhibitory effects of DRD2 on PRL secretion and ERK1/2 phosphorylation in primary cultured human PRL-secreting cells (18). Accordingly, DA-resistant PRL-secreting pituitary cells transfected with FLNA become able to respond to DAs (18).

FLNA is an homodimer of two subunits of 280 kDa each, containing an actin-binding domain at N-terminus, followed by 24 Ig-like repeats. The repeat 24 at the C-terminal acts as dimerization site and confers the protein its own characteristic ‘V’ shape (26). The main mechanism that regulates FLNA activity is the phosphorylation by protein kinase A (PKA) on Ser2152 residue, at the repeat 20 (27). This post translational modification affects the protein functions as it is involved in cell migration (28, 29), integrin binding (30), intracellular localization (29) and calpain cleavage (31).

In particular, the Ig19-20 region, which is involved in the formation of an autoinhibitory structure when FLNA is dephosphorylated (32), is implicated in the direct interaction with DRD2 (33). Therefore, it is eligible to suppose that PKA-induced FLNA phosphorylation (P-FLNA) might relate to its ability to bind DRD2 and its effectors, thus modulating DRD2 signal transduction.

In GH-secreting PitNETs, our group demonstrated that P-FLNA regulates SSTR2 signal transduction. We showed that phosphorylation switches FLNA function from a scaffold for signaling proteins, allowing SSTR2 signal transduction, to a signal termination protein that hampers all SSTR2 antitumoral effects, including inhibition of cell proliferation, migration and increase of cell apoptosis (25).

In the light of these premises, the aims of the present study were to evaluate in PRL- and ACTH-secreting pituitary tumor cell lines the modulation of FLNA phosphorylation by cAMP pathway activation and DRD2 agonist and to study the effects of P-FLNA on DRD2 intracellular signal transduction.

## Materials and Methods

### Pituitary Cell Cultures

Rat pituitary tumoral lactotroph MMQ cells (ATCC CRL-10609) were cultured in RPMI medium (Life Technologies, Thermo Fisher Scientific, Waltham, MA, USA) supplemented with 15% horse serum (HS), 2.5% fetal bovine serum (FBS), 2 mM glutamine and antibiotics (Gibco, Life Technologies, Thermo Fisher Scientific, Waltham, MA, USA). Murine pituitary tumoral corticotroph AtT-20 cells (ATCC CCL-89) were grown in F-10 medium (Life Technologies, Thermo Fisher Scientific, Waltham, MA, USA) supplemented with 10% FBS, 2 mM glutamine and antibiotics.

### Western Blot Analysis

Cells were incubated 10 min at 37°C with forskolin 1 μM (Sigma-Aldrich, St. Louis, MO, USA) or BIM53097 100 nM (kindly provided by Biomeasure Incorporated/IPSEN, Milford, MA, USA), while to perform time course experiments related times are indicated in the corresponding figures. For cyclin D3 and p27 expression levels analysis cells were treated with BIM53097 100 nM for 3 h and 48 h, respectively.

Total proteins extracted from cultured cells were quantified by BCA assay, separated on SDS/polyacrylamide gels and transferred to a nitrocellulose filter. Phospho-Filamin A (Ser2152), Filamin A, phospho-ERK1/2, ERK1/2, c-Myc and cyclin D3 antibodies (Cell Signaling Technology, Danvers, MA, USA) were used at 1:1,000. P27 and D2DR antibodies (Santa Cruz Biotechnology, Dallas, TX, USA) were diluted at 1:200. Phospho-AKT antibody was diluted at 1:2,000 and AKT at 1:1,000 (Immunological Science, Rome, IT). Primary antibodies were incubated overnight at 4°C, secondary antibodies anti-rabbit or anti-mouse (Cell Signaling Technology, Danvers, MA, USA) were used at 1:2,000 at room temperature for 1 h. Anti-GAPDH antibody (Ambion, Thermo Fisher Scientific, Waltham, MA, USA) was used at 1:4,000 for 1 h at room temperature. Chemiluminescence was detected trough a ChemiDOC-IT Imaging System (UVP, Upland, CA, USA) and then analysed with NIH ImageJ software.

### Plasmids Transfection

pcDNA3-Myc expression vectors coding for wild-type and S2152A FLNA were from Addgene (Watertown, MA, USA). S2152D FLNA was generated from the S2152A mutant as described previously (25). Briefly, the QuikChange XL SiteDirected Mutagenesis Kit (Agilent Techonologies, CA, USA) was employed to introduce a point mutation into S2152A FLNA cDNA. PCR-based mutagenesis, replacing Ala (GCA) with Asp (GAT), was performed using the following specific primers: reverse 5′– CAACGTTGGCCACATCAGGAGCCCGACGCC-3′ and forward

5′– GCAGGGGTCGGGCTCCTGATGTGGCCAACG-3’ (Invitrogen™, Thermo Fisher Scientific Carlsbad, CA, USA). Serine substitution with aspartic acid mimics phosphorylated FLNA, since the negative charge of the carboxyl group of aspartic acid mimics the negative charge of phosphate. On the contrary, serine substitution with alanine, an unphosphorylable amino acid, mimics dephosphorylated FLNA.

These vectors were transiently transfected in MMQ and AtT-20 cells for 72 h at 37°C using Lipofectamine 2000 reagent (Invitrogen, Thermo Fisher Scientific, Waltham, MA, USA), according to the instruction of the manufacturer. Transfection efficiency was monitored by Western blot analysis for each experiment by using an anti-c-Myc antibody (Cell Signaling Technology, Danvers, MA, USA). Empty vectors were used in each experiment as negative controls.

### Cell Proliferation Assay

Cell proliferation was assessed by colorimetric measurement of 5-bromo-2-deoxyuridine (BrdU) incorporation during DNA synthesis in proliferating cells according to the instruction of the manufacturer (GE Healthcare, Life Science, Buckinghamshire, UK), as previously described (34). MMQ and AtT-20 cells were seeded in starved medium in 96-well polylysine-coated plate at a density of 2 × 10^4^ cells/well. 24 h later, cells were transfected with plasmids for 72 h at 37°C, then incubated with or without BIM53097 100 nM at 37°C for 72 h (MMQ) or 96 h (AtT-20). The agonist was added the first day of the treatment for 72 h in both cell lines and added again the third day for 24 h in AtT-20 cells only (72 h + 24 h). BrdU incorporation in newly synthesized DNA was allowed for 2 h in MMQ and 24 h in AtT-20 cells. Each experiment was repeated four times and done in quadruplicate for each condition.

### Hormone Levels Detection

To detect prolactin (PRL) and adrenocorticotrophic (ACTH) hormone levels specific Elisa immunoassay kits (Fine Test, Wuhan Fine Biotech Co., Ltd, Wuhan, CN) were used.

MMQ and AtT-20 cells were seeded in six-well plate, at a density of 3,3 × 10^5^ cells/well. After 24 h cells were transfected with wild-type, S2152A and S2152D FLNA plasmids for 72 h at 37°C. Then cells were counted and re-seeded in a 24-well plate at a density of 12,5 × 10^4^ cells/well, in 300 μl of complete medium for AtT-20, while MMQ were re-seeded in a six-well plate at a density of 3,6 × 10^5^ cells/well, in 1 ml of complete medium. Cells were than incubated with or without BIM53097 100 nM for 4 h (AtT-20) or 24 h (MMQ), based on preliminary time course experiments. After treatment, culture media were collected to perform the assay, according to the manufacturer’s instructions. Absorbance was read at 450 nm in a Victor2 multilabel plate reader (Perkin Elmer, Whaltam, MA, USA). Data were plotted and analysed with the specific Curve Expert 1.4 program. Hormone detection were done in triplicate and experiments were replicated three times for each cell line. Hormone levels were normalized on the protein content, measured by BCA assay. Western blot analysis was carried out before each experiment to test transfection efficiency

### Statistical Analysis

The results are expressed as the mean ± S.D. A paired two-tailed Student’s t-test was used to detect the significance between two series of data. p < 0.05 was accepted as statistically significant.

## Results

### FLNA Phosphorylation Is Increased by PKA and Reduced by BIM53097 in PRL- and ACTH-Secreting Pituitary Cells

**Figure 1:**
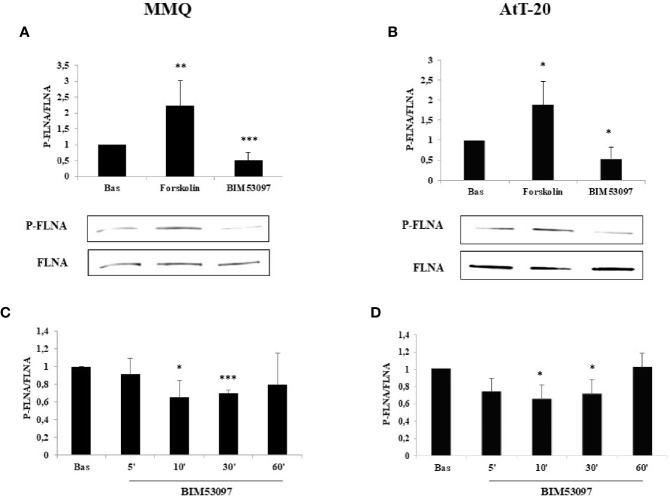
FLNA phosphorylation is reduced by DRD2 agonist and increased by forskolin treatment, respectively. MMQ cells **(A, C)** and AtT-20 cells **(B, D)** were treated with 1μM forskolin or 100 nM BIM53097 for 10 min **(A, B)** or indicated times **(C, D)** at 37°C. The graphs show the quantification of P-FLNA/total FLNA ratio. Experiments were repeated 5 times. Values represent mean ± S.D. normalized vs. respective basal. Representative immunoblots are shown. *p < 0.05, **p < 0.01, ***p < 0.001 vs. corresponding basal.

FLNA phosphorylation (P-FLNA) was tested by Western blot analysis in MMQ and AtT-20 cells. Our data showed that 10 min treatment with forskolin 1 μM determined an increase of FLNA phosphorylation levels on serine 2152 in both MMQ (+2.2 ± 0.8-fold, p < 0.01 vs. basal) (**Figure 1A**) and AtT-20 cells (+1.8 ± 0.58-fold, p < 0.05 vs. basal) (**Figure 1B**). On the other side, the incubation with DRD2 specific agonist BIM53097 100 nM induced a reduction of P-FLNA/FLNA ratio in both cell lines (-49.4 ± 25%, p < 0.001 vs. basal in MMQ; -45.8 ± 28%, p < 0.05 vs. basal in AtT-20) (**Figures 1A, B**). Moreover, time course experiments shown that agonist’s effect was significantly maintained until 30 min in both cell lines (**Figures 1C, D**).

### Phosphomimetic S2152D FLNA Mutant Reverted the Ability of BIM53097 to Reduce Cell Proliferation in Both MMQ and AtT-20 Cells

To analyze P-FLNA effects on DRD2 signal transduction, wild-type FLNA, phosphodeficient S2152A and phosphomimetic S2152D FLNA mutants were overexpressed in MMQ and AtT-20 cells, that endogenously express DRD2 (**Figure S1**).

**Figure 2:**
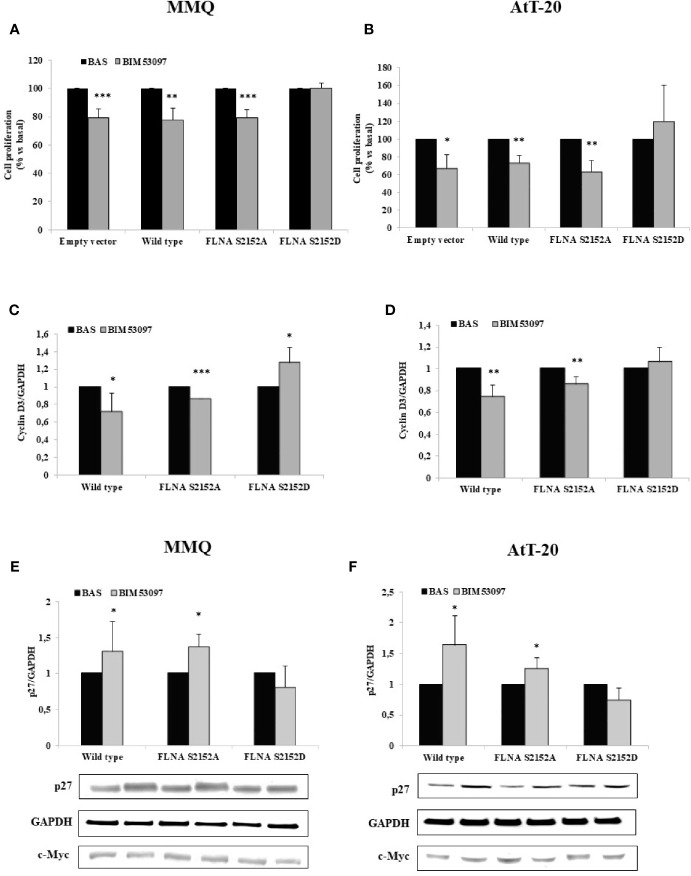
FLNA S2152D mutant reverted DRD2 inhibitory effects on cell proliferation in both MMQ and AtT-20 cells. MMQ **(A, C, E)** and AtT-20 cells **(B, D, F)** were transiently transfected with empty vector, wild-type, S2152A and S2152D FLNA mutants for 72 h at 37°C. MMQ **(A)** and AtT-20 **(B)** were than incubated 72 h or 96 h with 100 nM BIM53097, respectively. BrdU incorporation in newly synthesized DNA was measured. Experiments were repeated 4 times and each determination was done in quadruplicate. *p < 0.05, **p < 0.01, ***p < 0.001 vs. corresponding basal. **(C–F)** Cells were treated 3 h **(C, D)** or 48 h **(E, F)** with 100 nM BIM53097. The graphs show the quantification of cyclin D3 **(C, D)** or p27 **(E, F)** expression levels respectively, normalized to GAPDH. Representative immunoblots are shown. Data represent mean ± S.D. from three independent experiments. *p < 0.05, **p < 0.01, ***p < 0.001 vs. corresponding basal.

Proliferation assays measuring BrdU incorporation into *de novo* synthesized DNA revealed that the inhibitory effect exerted by BIM53097 in MMQ transfected with empty vector (-20.6 ± 6%, p < 0.001 vs. basal), was maintained in cells transfected with wild-type (-22.6 ± 8%, p < 0.01 vs. basal) and S2152A FLNA (-20.6 ± 5%, p < 0.001 vs. basal), but was abolished by phosphomimetic FLNA mutant overexpression (**Figure 2A**).

BIM53097 antimitotic effect was replicated in AtT-20 cells transfected with empty vector (-32.4 ± 15%, p < 0.05 vs. basal), wild-type (-26.3 ± 7%, p < 0.01 vs. basal) and S2152A FLNA (-36.6 ± 12%, p < 0.01 vs. basal), but was reverted by S2152D FLNA mutant overexpression (**Figure 2B**).

No significant differences were found in basal proliferation between empty vector, wild-type and FLNA mutants transfected cells (data not shown).

Moreover, cyclin D3 and p27 expression levels, markers of cell cycle, were then tested by Western blot analysis. In accordance, it emerged that the phosphomimetic FLNA mutant overexpression prevented the decrease of cyclin D3 expression induced by BIM53097 in wild-type and S2152A FLNA transfected MMQ (-28 ± 2%, p < 0.05 and -13.6 ± 0%, p < 0.001 vs. each basal, respectively) (**Figure 2C**) and AtT-20 cells (-25.8 ± 11%, p < 0.01 and -14.4 ± 6%, p < 0.01 vs. each basal, respectively) (**Figure 2D**). Consistently, the increase of p27 levels detected after DRD2 agonist treatment in wild-type and S2152A transfected MMQ (+1.3 ± 0.41-fold, p < 0.05 and +1.38 ± 0.17-fold, p < 0.05, vs. basal, respectively) and AtT-20 cells (+1.6 ± 0.40-fold, p < 0.05 and +1.25 ± 0.17-fold, p < 0.05, vs. basal, respectively), was not observed after S2152D FLNA transfection (**Figures 2E, F**). BIM53097 effects on cyclin D3 (**Figures S2A, B**) and p27 (**Figures S3A, B**) in empty vector transfected cells were similar to those measured in cells expressing wild-type FLNA.

### Effects of S2152D FLNA Mutant Overexpression on ERK1/2 and AKT Phosphorylation

**Figure 3:**
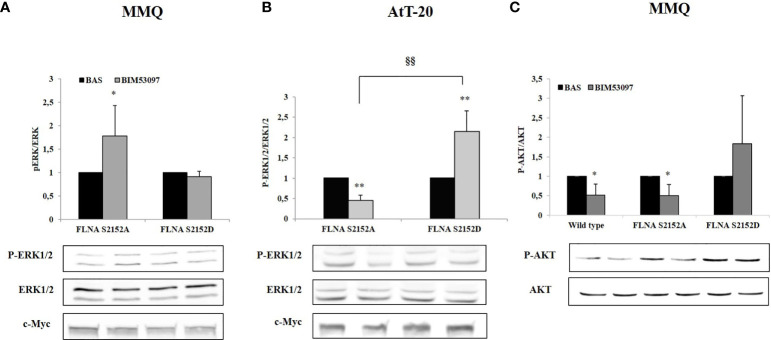
Effects of S2152D FLNA mutant overexpression on ERK1/2 and AKT phosphorylation. MMQ **(A, C)** and AtT-20 cells **(B)** were treated 10 min with 100 nM BIM53097. The graphs show the quantification of P-ERK1/2 expression levels normalized to total ERK1/2 (**A** and **B**) and P-AKT/total AKT **(C)**. Data represent mean ± S.D. from three independent experiments. Representative immunoblots are shown. *p < 0.05, **p < 0.01 vs. corresponding basal. ^§§^p < 0.01 vs. corresponding BIM53097 treated.

In agreement, a significant increase of ERK1/2 phosphorylation levels after BIM53097 incubation was observed in S2152A FLNA expressing MMQ cells (+1.8 ± 0.6-fold, p < 0.05 vs. basal), but this effect was abolished when cells were transfected with S2152D FLNA mutant (**Figure 3A**). Concordant results were obtained in AtT-20 cells with expected ERK1/2 decreased activity (-0.55 ± 0.13-fold, p < 0.01 vs. basal) in phosphodeficient FLNA expressing cells (**Figure 3B**).

Since BIM53097 reduced AKT phosphorylation/total AKT ratio in MMQ cells, we tested P- FLNA effects on this key modulator. BIM53097 treatment determined a reduced phosphorylation of AKT in wild-type (-48.7 ± 28%, p < 0.05 vs. basal) and S2152A (-49 ± 28%, p < 0.05 vs. basal) FLNA expressing cells, effect that was abolished when overexpressing phosphomimetic mutant (**Figure 3C**).

Conversely, DRD2 agonist incubation did not induce a reduction of AKT phosphorylation levels in AtT-20 cells (data not shown).

### FLNA Phosphorylation Reverted the Ability of BIM53097 to Reduce PRL and ACTH Secretion in MMQ and AtT-20 Cells, Respectively

Phosphodeficient S2152A and phosphomimetic S2152D FLNA mutants were overexpressed in MMQ and AtT-20 cells to test their effects on cells hormonal secretion.

**Figure 4:**
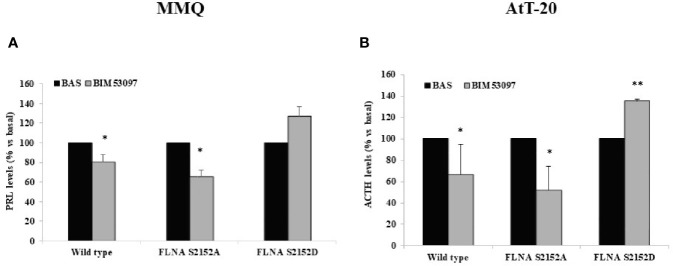
FLNA phosphorylation abolished antisecretory effects of DRD2. MMQ **(A)** and AtT-20 cells **(B)** were transiently transfected with wild-type, S2152A and S2152D FLNA mutants for 72 h at 37°C. After 4 h (MMQ) or 24 h (AtT-20) BIM53097 treatment, hormonal assay was performed in order to detect hormones levels. The graphs show the percentage of PRL **(A)** and ACTH **(B)** secretion levels. Data represent mean ± S.D. from three independent experiments and each determination was done in triplicate. *p < 0.05, **p < 0.01 vs. corresponding basal.

Our results revealed that BIM53097 induced a reduction of hormonal secretion in wild-type (-20 ± 10%, p < 0.05 in MMQ, -33.3 ± 23%, p < 0.05 in AtT-20 vs. each basal) and S2151A (-34.3 ± 6%, p < 0.05 in MMQ, -48.2 ± 22%, p < 0.05 in AtT-20 vs. each basal) FLNA transfected cells in both pituitary cell lines. Furthermore, phosphomimetic mutant reverted the anti-secretory ability of DRD2 agonist on PRL and ACTH release by MMQ and AtT-20, respectively (**Figures 4A, B**).

## Discussion

To date, the molecular mechanisms underlying drug resistance of PitNETs have not been fully understood and no biomarkers predictable of response to pharmacological treatment are yet available. In the recent past a relevant role for cytoskeleton in regulating receptor dynamics was highlighted. The actin binding protein FLNA emerged as important player in the regulation of the complex intracellular processes that dictate pituitary tumors drug responsiveness and invasiveness. It participates in actin filaments crosslinking and in mediating somatostatin and dopamine receptors intracellular effects.

In particular, our group previously demonstrated FLNA essential tasks in mediating SSTR2 and DRD2 expression, intracellular localization and signaling in GH- and PRL-secreting PitNETs, respectively, playing a role in responsiveness to somatostatin analogs and dopaminergic drugs (18, 25). Moreover, a latest study positively correlated FLNA and DRD2 expression in somatotropinomas (35), in line with our previous report in MMQ cell line (18). On the other hand, DRD2 expression was not associated with FLNA expression nor to the response to cabergoline treatment in corticotrophinomas and FLNA expression, in turn, did not correlate with dopamine agonist effect (36).

However, the evaluation of FLNA protein expression does not provide information about its activity. This last is regulated by several mechanisms, including phosphorylation, mechanical force, intramolecular inhibition, competition with other molecules, alternative splicing and proteolysis (26). FLNA is substrate of phosphorylation of different kinases, among which PKA, that phosphorylates it on the residue S2152, located in Ig20, with important effects on its conformation and activity (25).

DRD2 is the main target for pharmacological therapy with DAs in prolactinomas and different studies indicated it as a potential target also for other DRD2-expressing tumors, such as ACTH-secreting PitNETs, tough there is still a lack in clinical evidences. For these reasons in this study we investigated FLNA phosphorylation (P-FLNA) effects on DRD2 signal transduction in PRL- and ACTH-secreting PitNETs. Our experiments were conducted in two tumoral pituitary cell lines both endogenously expressing DRD2: rat lactotrophs, MMQ, and murine adrenocorticotrophs, AtT-20.

First of all, we showed that FLNA phosphorylation status is regulated by cAMP/PKA. In agreement with previous data obtained *in vitro* on pituitary tumoral somatotrophs (25) we showed that cAMP/PKA pathway activation induced FLNA phosphorylation at Ser2152 also in pituitary tumoral lactotrophs and adrenocorticotrophs. Indeed, forskolin increased P-FLNA/FLNA ratio and the DRD2 selective agonist, BIM53097, significantly reduced P-FLNA in both cell lines.

To better understand the effects of Ser2152 FLNA phosphorylation on DRD2 signal transduction we transfected phosphodeficient S2152A or phosphomimetic S2152D FLNA mutant in MMQ and AtT-20 cells.

The main pathways involved in cell growth and proliferation regulation are MAPK and PI3K/AKT ones. According to literature, we confirmed a DA-induced increased phosphorylation of ERK1/2 in tumoral lactotrophs, that leads to cell proliferation inhibition (18, 37, 38). On the other side, BIM53097 treatment determined a reduction of ERK1/2 phosphorylation levels. This result is in line with literature where a reduction of ERK1/2 activity has been associated yet with a reduction of cell proliferation in AtT-20 (39).

FLNA phosphorylation abolished its endogenous functions, in fact, from our results emerged that DRD2 agonist antiproliferative effects were abrogated when S2152D FLNA was overexpressed in both cell models.

DRD2 is able to reduce AKT phosphorylation levels in tumoral lactotrophs (38, 40), and we recently demonstrated that BIM53097 determined a significant reduction of AKT activation in MMQ cells and in a subset of NF-PitNETs through a β-arrestin 2-mediated mechanism (41).

In the present study, we showed that phosphomimetic FLNA mutant reverted the ability of BIM53097 to reduce AKT phosphorylation levels in MMQ cells, suggesting that P-FLNA might also influence DRD2 binding to partner molecules, but additional investigations are certainly required in order to elucidate molecular mechanism involved.

Moreover, the well-established capability of DAs of reducing PRL levels in tumoral lactotrophs (42, 43) was maintained in wild-type and phosphodeficient FLNA mutant transfected cells, but was abrogated by phosphomimetic one.

Accordingly, as pointed out in different studies, even if in variable way (5, 44, 45), DAs induced an inhibition of ACTH secretion in tumoral corticotrophs *via* DRD2 activation, effect that we showed to be impaired by S2152D FLNA mutant overexpression.

We must put in evidence that wild-type and S2152A FLNA overexpression did not affect DRD2 effects induced by DRD2-agonist incubation. This point is in line with FLNA scaffold role, since it has been demonstrated that the overexpression of a scaffold protein does not necessarily intensify signaling (46).

Furthermore, a key role of phosphorylation in regulating FLNA binding properties has been already shown, in particular that phosphorylation on Ser2152 induces integrin binding to Ig21 leading to the separation of the autoinhibitory structure that involves Ig19, Ig20 and Ig21 (30, 47). It has also been demonstrated that PKA-dependent FLNA phosphorylation could be allowed by disruption of Ig21 induced by FLNA rearrangement due to ligands recruitment (32).

In our previous study (25) we partially clarified the molecular mechanism by which P-FLNA disrupted SSTR2 signaling. Indeed, trough co-immunoprecipitation experiments we showed a reduction of G_i1-2-3_ binding to SSTR2 in S2152D FLNA transfected cells with respect to those overexpressing S2152A FLNA. In light of these data, we can hypothesize that a similar mechanism occurred with DRD2. As previous demonstrated FLNA plays a key role in regulating DRD2 expression and signaling in PRL-secreting PitNETs. Indeed, it is required for DRD2-mediated ERK1/2 activation, inhibition of PRL release and cell proliferation (18). Further studies are needed to identify which molecular mediators involved in the DRD2 signaling cascade are affected by FLNA phosphorylation.

To the best of our knowledge, currently there are no other evidences of P-FLNA as key modulator of GPCRs signaling in PitNETs in addition to our previous on somatotrophs (25), or in other tumors as well. We can speculate that phosphorylation might represent a novel regulatory mechanism shared by different GPCRs that switch FLNA function from a scaffold enabling receptor signal transduction to a signal termination protein.

Admittedly, a limitation of our study is the absence of primary cultures of human PRL- or ACTH-secreting PitNETs. Further studies are needed to confirm and strengthen these results in human cells.

In conclusion, our data showed that cAMP pathway and DRD2 agonist regulated FLNA activity by increasing or decreasing, respectively, its phosphorylation on Ser2152. This study additionally turned out that FLNA phosphorylation prevented DRD2 signaling in PRL- and ACTH-secreting tumoral pituitary cell lines. In PitNETs expressing DRD2, modulation of P-FLNA might suggest new pharmacological strategies to overcome drug resistance and P-FLNA might represent a new molecular biomarker for tumor responsiveness to DAs.

## Data Availability Statement

The raw data supporting the conclusions of this article will be made available by the authors, without undue reservation.

## Author Contributions

FM: Conceptualization, methodology, investigation, data curation, writing (original draft), writing (review and editing), and formal analysis. DT, RC, GiuM, and GDM: Investigation. AS and MA: Review and editing. EP: Conceptualization, validation, data curation, funding acquisition, supervision, writing (original draft), writing (review and editing), project administration, formal analysis. GioM: Conceptualization, supervision, funding acquisition, project administration, and writing (review and editing). All authors contributed to the article and approved the submitted version.

## Funding

This work was supported by AIRC (Associazione Italiana Ricerca Cancro) grant to GioM (IG 2017-20594), Italian Ministry of Health grant to GioM (PE-2016-02361797), Ricerca Corrente Funds from the Italian Ministry of Health, and Progetti di Ricerca di Interesse Nazionale (PRIN) grant to EP (2017N8CK4K).

## Conflict of Interest

The authors declare that the research was conducted in the absence of any commercial or financial relationships that could be construed as a potential conflict of interest.
