# Supplementary figures and images for: A Novel Mechanism Regulating Dopamine Receptor Type 2 Signal Transduction in Pituitary Tumoral Cells: The Role of cAMP/PKA-Induced Filamin A Phosphorylation
Source: Front Endocrinol (Lausanne). 2021 Feb 16;11:611752. doi: 10.3389/fendo.2020.611752 (PMC7921166; doi:10.3389/fendo.2020.611752)

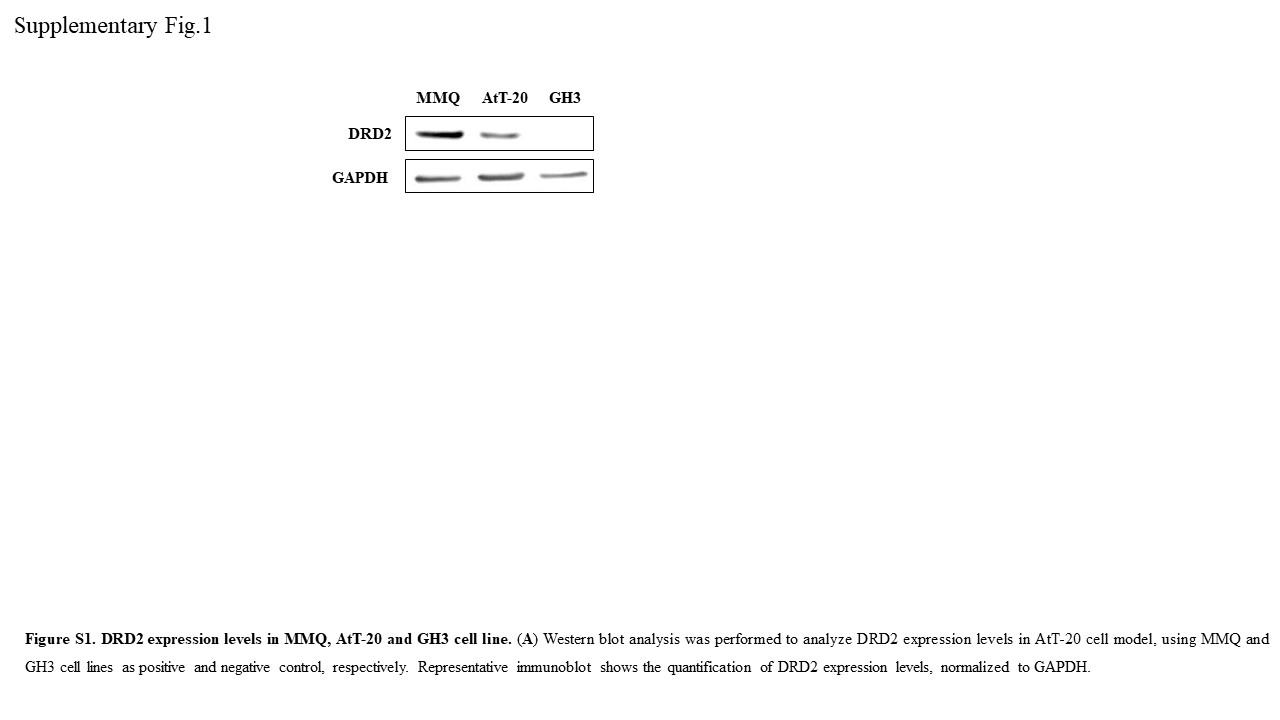

Supplement: Supplementary file 1 [file Image_1.jpeg]

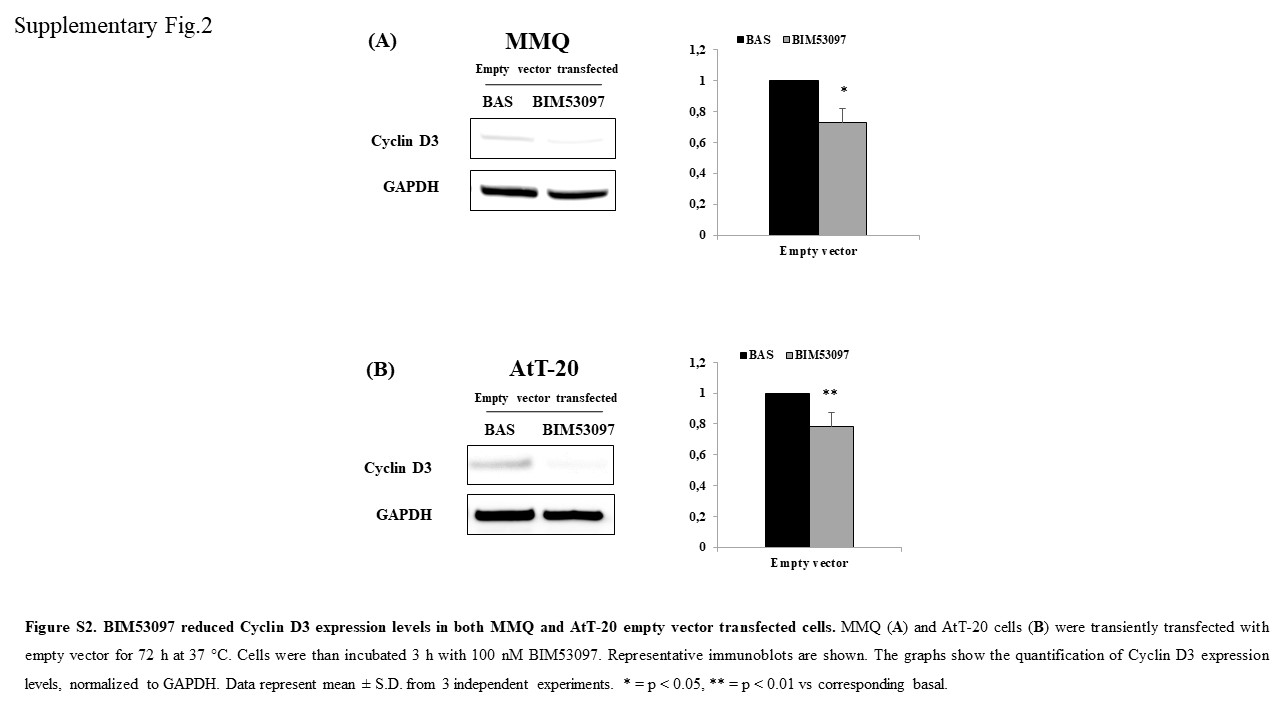

Supplement: Supplementary file 2 [file Image_2.jpeg]

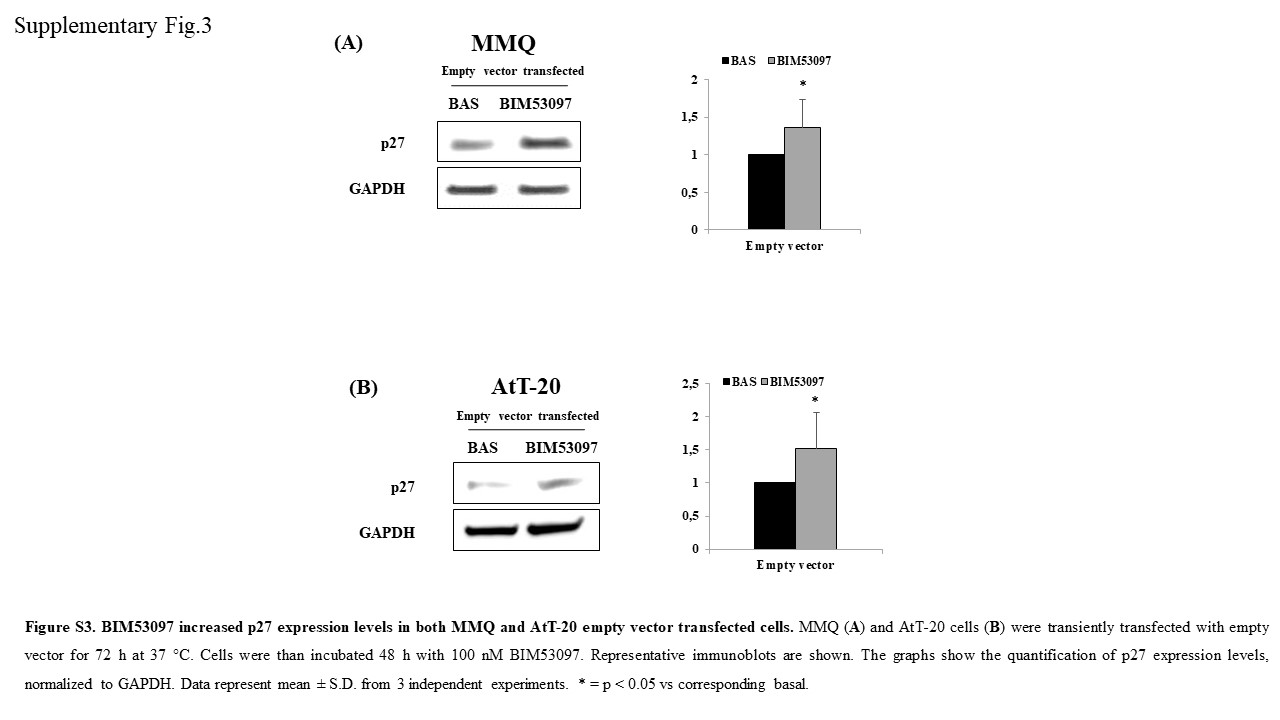

Supplement: Supplementary file 3 [file Image_3.jpeg]
